# Myocardial Iron Overload in an Experimental Model of Sudden Unexpected Death in Epilepsy

**DOI:** 10.3389/fneur.2021.609236

**Published:** 2021-02-11

**Authors:** Enes Akyuz, Zuleyha Doganyigit, Ece Eroglu, Franco Moscovicz, Amalia Merelli, Alberto Lazarowski, Jerónimo Auzmendi

**Affiliations:** ^1^Department of Biophysics, Medical School, Yozgat Bozok University, Yozgat, Turkey; ^2^Department of Histology and Embryology, Medical School, Yozgat Bozok University, Yozgat, Turkey; ^3^Medical School, Yozgat Bozok University, Yozgat, Turkey; ^4^Department of Clinical Biochemistry, School of Pharmacy and Biochemistry, Pathophysiology and Clinical Biochemistry Institute (INFIBIOC), University of Buenos Aires, Buenos Aires, Argentina; ^5^National Council of Science and Technology (CONICET), Buenos Aires, Argentina

**Keywords:** SUDEP, epilepsy, heart failure, iron overload cardiomyopathy, ferroptosis

## Abstract

Uncontrolled repetitive generalized tonic-clonic seizures (GTCS) are the main risk factor for sudden unexpected death in epilepsy (SUDEP). GTCS can be observed in models such as Pentylenetetrazole kindling (PTZ-K) or pilocarpine-induced Status Epilepticus (SE-P), which share similar alterations in cardiac function, with a high risk of SUDEP. Terminal cardiac arrhythmia in SUDEP can develop as a result of a high rate of hypoxic stress-induced by convulsions with excessive sympathetic overstimulation that triggers a neurocardiogenic injury, recently defined as “Epileptic Heart” and characterized by heart rhythm disturbances, such as bradycardia and lengthening of the QT interval. Recently, an iron overload-dependent form of non-apoptotic cell death called ferroptosis was described at the brain level in both the PTZ-K and SE-P experimental models. However, seizure-related cardiac ferroptosis has not yet been reported. Iron overload cardiomyopathy (IOC) results from the accumulation of iron in the myocardium, with high production of reactive oxygen species (ROS), lipid peroxidation, and accumulation of hemosiderin as the final biomarker related to cardiomyocyte ferroptosis. Iron overload cardiomyopathy is the leading cause of death in patients with iron overload secondary to chronic blood transfusion therapy; it is also described in hereditary hemochromatosis. GTCS, through repeated hypoxic stress, can increase ROS production in the heart and cause cardiomyocyte ferroptosis. We hypothesized that iron accumulation in the “Epileptic Heart” could be associated with a terminal cardiac arrhythmia described in the IOC and the development of state-potentially in the development of SUDEP. Using the aforementioned PTZ-K and SE-P experimental models, after SUDEP-related repetitive GTCS, we observed an increase in the cardiac expression of hypoxic inducible factor 1α, indicating hypoxic-ischemic damage, and both necrotic cells and hemorrhagic areas were related to the possible hemosiderin production in the PTZ-K model. Furthermore, we demonstrated for the first time an accumulation of hemosiderin in the heart in the SE-P model. These results suggest that uncontrolled recurrent seizures, as described in refractory epilepsy, can give rise to high hypoxic stress in the heart, thus inducing hemosiderin accumulation as in IOC, and can act as an underlying hidden mechanism contributing to the development of a terminal cardiac arrhythmia in SUDEP. Because iron accumulation in tissues can be detected by non-invasive imaging methods, cardiac iron overload in refractory epilepsy patients could be treated with chelation therapy to reduce the risk of SUDEP.

## Introduction

Sudden unexpected death in epilepsy (SUDEP) is the single leading cause of premature death in people with epilepsy, affecting ~1 in 1,000 patients (1). One of the most important clinical risk factors related to SUDEP is a high frequency of uncontrolled generalized tonic-clonic seizures (GTCS) that result in an increased risk of death in patients with refractory epilepsy (2–4).

Although the mechanism that triggers sudden death is unknown, several lines of evidence suggest that SUDEP could be due to a terminal cardiac arrhythmia as a consequence of a highly frequent seizure-triggered hypoxic-stress plus excessive sympathetic overstimulation that triggers neurocardiogenic injury and affects electrical properties of the myocardium resulting in a propensity for severe bradycardia, and fatal cardiac arrhythmia (5). In this sense, the alteration in cardiomyocytes such as vacuolization and nucleus displacement by vacuole was described as a reversible pathological condition.

However, diffuse interstitial fibrosis with multiple areas of cardiomyocyte replacement by connective tissue and perivascular fibrosis were also observed and interpreted as irreversible pathological changes (6). Although, cardiac fibrosis was reported to be more frequently detected in SUDEP cases, a more recent study demonstrated that this cardiac pathology was less severe in SUDEP cases than sudden arrhythmic death (7). The lower level of cardiac damage observed in SUDEP suggests that sudden death is not primarily related to anatomical abnormalities but to functional abnormalities secondary to chronic and persistent hypoxic stress. In this way, exceeding a cumulative stress threshold, a further seizure could trigger a fatal cardiac arrhythmia.

In this regard, autonomic cardiac neural discharges have been characterized after pentylenetetrazol (PTZ)-induced epileptogenic activity (8). In this sense, cardiac hypertrophy seems to be related to the autonomic changes that arise from seizures, suggesting that the heart could be structurally affected, as observed in a chronic epileptic rat model (9). Structural cardiac pathologies can provide more insight into the SUDEP heterogeneous mechanisms (10).

Experimental data obtained from a Dravet Syndrome model showed that cardiomyocytes exhibited increased excitability, prolonged action potential duration, and triggered activity. In this model, continuous radiotelemetric electrocardiographic (ECG) recordings showed QT-interval (QT) prolongation, ventricular ectopic foci, idioventricular rhythms, beat-to-beat variability, ventricular fibrillation, and focal bradycardia that were associated with spontaneous deaths in mice (11). Accordingly, QT prolongation and heart rate alteration were reported after a single pilocarpine-induced status epilepticus (SE) in rats, whereas subsequent SE was associated with an increased SUDEP frequency and a worsening of electrophysiology (12). Furthermore, upregulation of P-glycoprotien (P-gp) and downregulation of inward-rectifier potassium channels (Kir) have been proposed to alter the electrical properties and contractibility of cardiomyocytes (13, 14). It has recently been demonstrated that the expression of ubiquitin-specific peptidase 15 (USP15) was upregulated in a PTZ-kindled (PTZ-K) rat model of epilepsy. USP15 upregulation was associated with increased levels of intracellular reactive oxygen species (ROS) and enhanced superoxide dismutase (SOD) activity (15). SOD, which converts superoxide (${\text{O}}_{2}^{-}$) into hydrogen peroxide (H_2_O_2_) and dioxygen (O_2_), can act through a reaction called disproportionation as the front line of defense against ROS-mediated injuries (16).

Oxidative stress is assumed to be a major factor in IOC. Reduced free iron ions (Fe^2+^) participate in the ROS formation via Haber-Weiss and Fenton reactions. Because cardiac tissue contains high levels of functional L-type voltage-gated Ca^2+^ channels, hypoxic conditions can turn cardiac tissue especially susceptible to free iron overload (17, 18). Additionally, divalent transporters such as the zinc transporter ZIP_8_ and ZIP_14_ provide an active pathway for Fe^2+^ entry that can lead to multiple deleterious effects (19, 20). In a hypoxia-inducible factor 1α (HIF-1α)-dependent manner, hypoxia will also upregulate the expression of transferrin receptor (Tfr-R) through which more iron will enter the cell, resulting in various structural damage and functional alterations, such as impaired excitation contraction coupling. Furthermore, impaired relaxation and subsequent contractility, lipid peroxidation, mitochondrial dysfunction with reduced ATP production, and direct DNA damage can be observed (17, 21). In this regard, a feedback mechanism has been described between the participation of oxidative stress with inflammation and excitotoxicity in refractory epilepsy (22, 23). We suspect that those could also be present in the heart as a consequence of repetitive and/or severe seizures.

These functional changes were also described in conditions of cardiac hypoxia, where the oxidative environment promotes iron deposition that is associated with a slow mechanism of cell death called ferroptosis (24). We hypothesize that in refractory epilepsy, recurrent episodes of hypoxia and oxidative stress will induce a progressive accumulation of iron in cardiomyocytes as a ferroptosis hallmark of Iron Overload Cardiomyopathy (IOC) and propensity to terminal cardiac arrhythmia associated with SUDEP. The aim of this study was to evaluate the presence of hemosiderin deposits in the heart in an experimental model of SUDEP secondary to SE.

## Materials and Methods

### Experimental Model

#### Pentylenetetrazole Kindling Model

The PTZ-K epilepsy model was induced using an established protocol (25). The rats were injected intraperitoneally with a subconvulsive dose of PTZ (35 mg/kg) three times a week (Monday, Wednesday, and Friday) for 1 month until kindling occurred. After each injection, the rats were placed alone in an isolated transparent Plexiglas cage and their convulsive behavior was independently monitored for 30 min. Records were blindly analyzed to determine seizure stage, onset latency, and duration. Seizure severity was scored using Racine's classification (25) as follows: no response (Score 0); facial movements, ear, and whisker twitching (Score 1); myoclonic convulsions without rearing (Score 2); myoclonic jerks, upright position with clonic forelimb convulsions (Score 3); clonic-tonic convulsions (Score 4); generalized clonic-tonic seizures with loss of postural control (Score 5) and finally death (Score 6).

#### Lithium-Pilocarpine Paradigm

SE was induced weekly for 3 weeks as previously described (12). Briefly, 200–250 g male Wistar rats were maintained at 20–25°C, 60% humidity, 12/12 h light/dark cycle, and food *ad libitum*. Rats were administered lithium chloride (33 mEq/kg i.p.) 18 h prior to pilocarpine treatment (30 mg/kg i.p.). Convulsive behavior was assessed using the Racine scale described above and only animals that were kept for at least 5 min with GTCS were included in this study. SE was limited with diazepam (20 mg/kg i.p.) at 20 min. All rats were supplied with 1 ml of H_2_O i.p. every 12 h. The rats were allowed recovery periods of 48 h after SE in which vital signs were closely monitored.

### Tissue Preparation

#### Fixation and Cryostat Preservation

Animals exposed to the pilocarpine paradigm were routinely fixed as previously described (12). Briefly, the rats were anesthetized with ketamine/xylazine (k/x: 90/10 mg/kg, i.p.). After thoracic surgery, the heart was exposed and the blood was then flushed by perfusion from the left ventricle using a 21G butterfly needle. Reverse circulation was achieved by cutting the right atrium and then applying 60 ml of saline plus heparin (50 UI/ml). A first fixation step was carried out applying 60 ml of 4% paraformaldehyde (PFA) in cold phosphate buffer saline (PBS). The final fixation step was performed using 300 ml of 4%PFA applied by gravity. After fixation, hearts were removed and fixed after 12 h and then washed with PBS. Finally, the hearts were snap-frozen and stored at −20°C until cryostat processing. Frozen hearts were serially cut into 30-micron coronal sections and then placed into one of 20 slices each. In this way, each slice contains a section separated by 600 microns.

#### Fixation and Paraffin Inclusion

Tissue samples obtained from rats exposed to the kindling model were determined with a 10% formaldehyde solution for use in histological examinations. After the detection process, routine tissue follow-up steps were applied to the tissues and embedded in paraffin to perform the paraffin blocks (10).

### Cytochemistry

#### Perls Staining

Slices containing a 30 μm thick frozen section at the coronal incidence were used for iron deposition determination. One out of 10 consecutive sections was stained and analyzed for hemosiderin aggregates. Briefly, the slices were incubated with a solution of 10 g/l potassium ferrocyanide in 0.1 mol/l HCl for 10 min at room temperature. Then the slices were washed well under running tap water for at least 20 min. The slices were contrast-stained using a dilute safranin solution (1% v/v) for 1 min. To prevent false-positive Perls staining, all materials used in this technique were washed with 1 M HCl for 24 h.

#### Hematoxylin-Eosin Staining

Tissue sections (5 μm) were obtained from the paraffin blocks and then placed on polylysine-coated slices for morphological analysis. The prepared slides were left in the oven for a certain period using standard histological methods as described in Akyuz et al. (10). Xylene and paraffin were removed and passed through a graded alcohol series and diluted. After being covered with Entellan® (107,960 Sigma-Aldrich), cardiac tissues were examined with the Olympus BX53 microscope. With the ImageJ program, 25 cell lengths from each group were measured.

### Immunohistochemistry

The Avidin-Biotin-peroxidase method was used immunohistochemically for the determination of cardiac anti-HIF-1α ([H1alpha67] (ab1) Abcam) immunoreactivity. The 5 μm thick sections taken from paraffin blocks were kept in an oven at 60°C overnight and then passed through xylene and decreasing graded alcohol series. Then, after washing with distilled water, it was treated with citrate buffer for antigen recovery. Hydrogen peroxide was applied after washing with PBS and the Large Volume Detection System kit (NB100-479, Novus Biologicals, USA) was used. After that, serum block application was performed for 5 min. After blocking the serum, HIF-1α was applied at 4°C overnight as the primary antibody. Secondary streptavidin-HRP (TP-125-HL; Thermo Scientific) and DAB chromogen with Biotin were applied, contrast staining with Gill Hematoxylin. Finally, it was passed through the gradually increasing alcohol-xylene series and closed with Entellan. Images obtained with the DP71 digital camera under the Olympus BX53 brand light microscope were analyzed for image differences using Image J 1.48v software.

### Data and Statistical Analysis

The free software of the National Institute of Health Image-J was used to measure the immunoreactivity of 25 areas of the images taken from the 5 preparations of each group. In the same way, the length of 25 cardiomyocytes per group was analyzed. The positive Perls reactions of 15–30 non-consecutive coronal sections were analyzed for each heart in the control and three SE groups. The Graphpad Prism 8.0 program was used for statistical analysis. Results are shown as the mean standard deviation. The independent sample *T*-test was used for intergroup comparison. A statistical value of *p* < 0.05 was considered significant.

## Results

### Pentylenetetrazol Kindling Model

**Figure 1:**
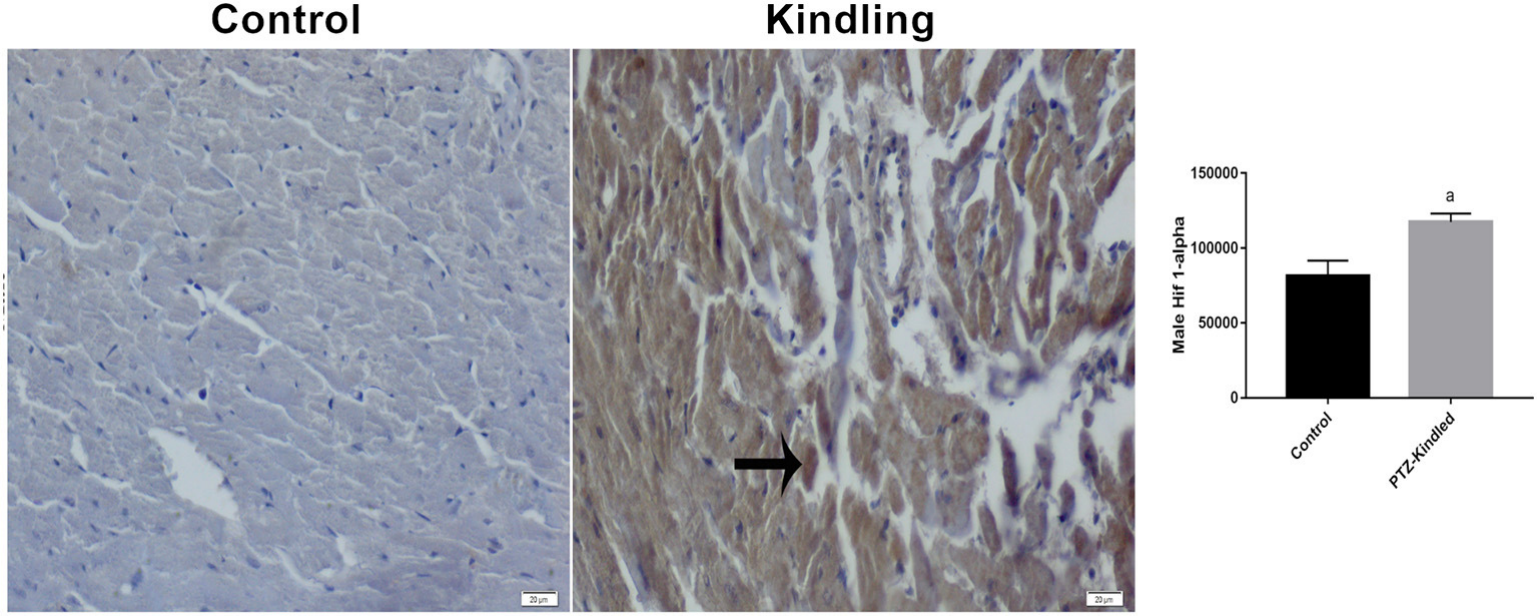
Hif 1-α immunoreactivity staining images and graphics. Immunoreactive areas are shown with a black arrow. Compared with the control, ^a^*p* < 0.05 was considered significant. x400.

After the 13th PTZ injection, all rats developed chronic epilepsy-like behavior with spontaneous generalized tonic-clonic seizures (stage 5). The mean degree of phase of all injections was 5.31 ± 0.58 and the duration of the seizures was 308 ± 95 s. Moreover, we did not observe a significant difference in body weight between the corresponding experimental group. Cardiomyocytes showed a clear difference in HIF-1α immunoreactivity between the control group and the kindled group. The HIF-1α immunoreactivity increased in the cardiomyocytes from 81.58 ± 10.12 μm^2^ -control- to 117.39 ± 5.69 μm^2^ -kindling- (*p* = 0.0001) (Figure 1). This result is in the same way as our previous report on HIF-1α immunoreactivity in cardiomyocytes under SE (12).

**Figure 2:**
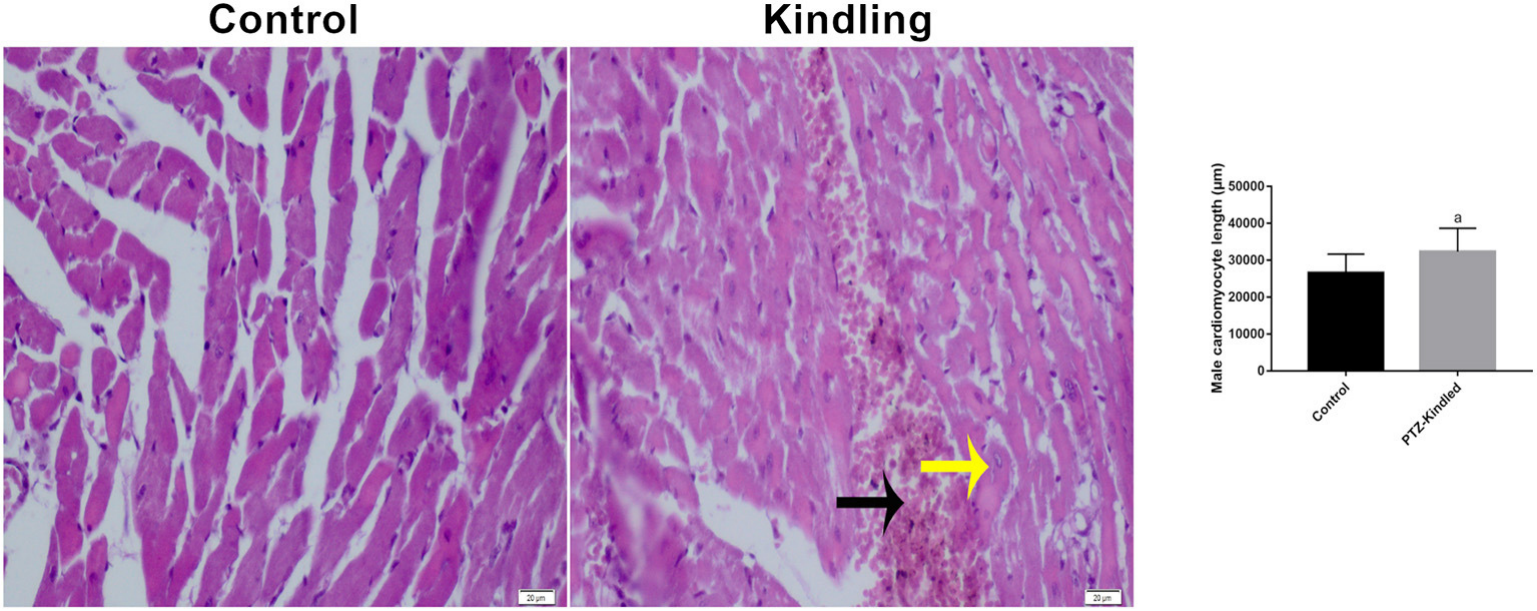
Plots of Hematoxylin-eosin staining image and cardiomyocyte length. Necrotic cells are shown with a yellow arrow and hemorrhagic areas with a black arrow. Compared with the control ^a^*p* < 0.05 was considered significant.

Furthermore, we found morphological differences between the cardiomyocytes of the controls and the kindled rats (Figure 2). PTZ treatment increased cardiomyocyte length by ~20% (26.60 ± 5.02 μm -control- vs. 32.29 ± 6.35 μm - kindled - *p* = 0.003). Additionally, in the rat heart of the PTZ-K model, large hemorrhagic areas (Figure 2 black arrows) and necrotic cells (Figure 2 yellow arrows) were observed. Interestingly, both morphological alterations are related to iron deposits such as hemosiderin accumulation (26).

### Status Epilepticus Induced by the Pilocarpine Paradigm

**Figure 3:**
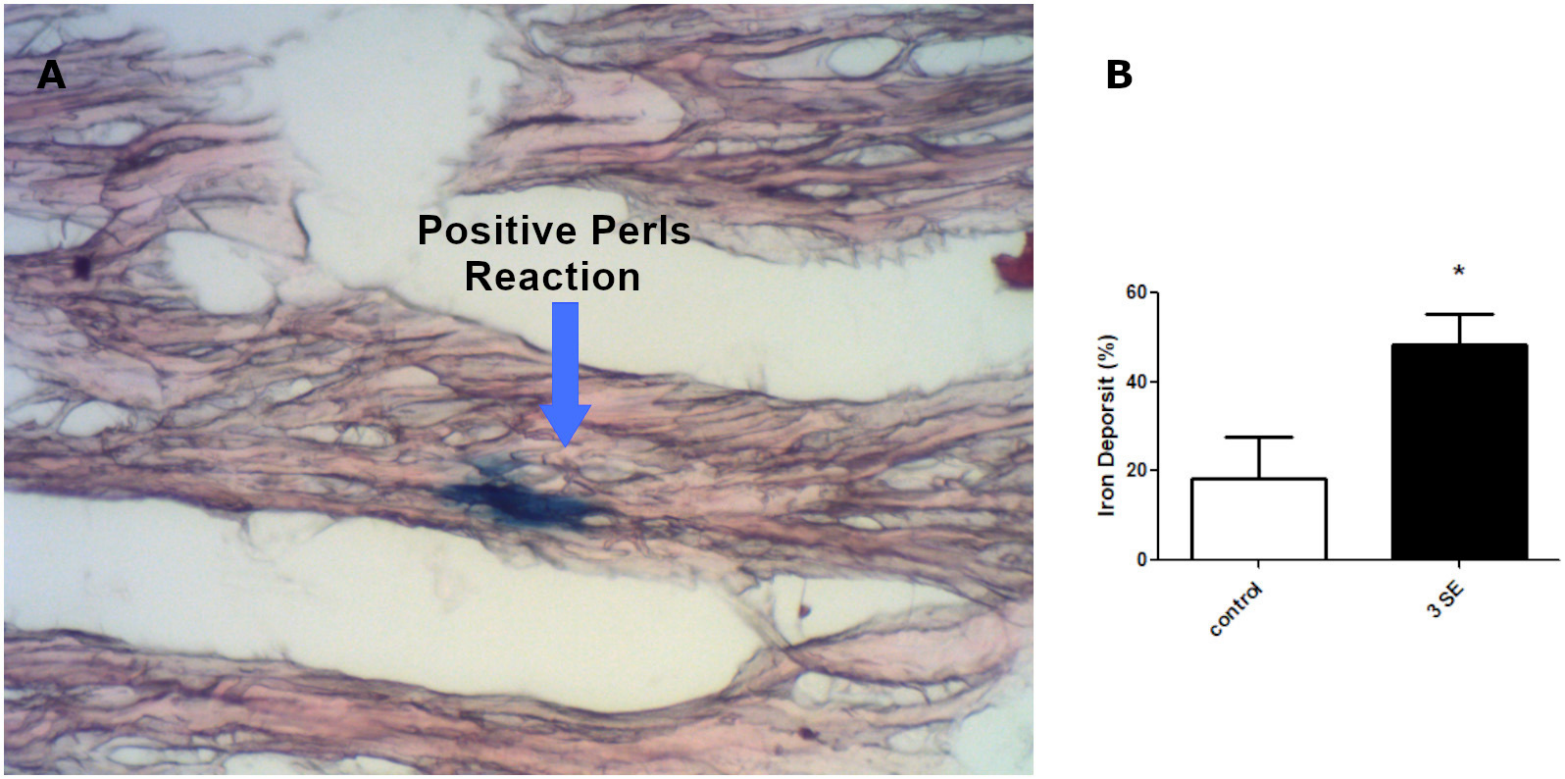
Iron deposition in cardiomyocytes. **(A)** Micrograph at x400 showing cardiomyocites (red) and iron deposition (blue). **(B)** Quantitation of positive iron deposition in myocardial coronal sections of four rats after the third SE *p < 0.05.

Taking into account that the kindling model induces hypoxic cardiac stress similar to that we previously reported under SE (12), we analyzed the iron deposition in the heart after severe convulsive stress produced by three consecutive weekly-induced SE. We used Perls staining to visualize the iron deposit as a blue precipitate (Figure 3) and found 2.5 folds that increased a positive blue mark (18.33% ±9.28 -control- vs. 48.39% ±6.73−3 SE- *p* = 0.0427).

## Discussion

Sudden unexpected death in epilepsy is the most important cause of death in people with epilepsy. Although different conditions that predispose to SUDEP have been described, clinical and experimental studies show that high frequency of GTCS is the most important risk factor, which explains why refractory epilepsies have a higher risk of SUDEP. Despite the fact that SUDEP is assumed to be a neurocardiorespiratory injury, the intrinsic mechanism that triggers sudden death is still unknown. Additionally, obstructive apnea has also been proposed as a key factor because of ictal involvement of brainstem structures producing laryngospasm (27, 28). In this sense, it has recently been reported that an oxygen-rich atmosphere, as well as selective serotonergic reuptake inhibitors, could prevent seizure-associated death and lead to a recovery of respiratory function after the seizure in mice (29). In association with these experimental data, a clinical study called MORTEMUS (Mortality Monitoring Unit Study in Epilepsy) suggests that the potential SUDEP mechanism could be centrally mediated and, therefore, alter respiratory and cardiac functions as a consequence of GTCS (30). Furthermore, some controversial concepts between SUDEP and sudden cardiac death during epilepsy have yet to be resolved.

In a previous report we described for the first time, a direct relationship between repetitive seizures with progressive P-gp overexpression in the heart and brain parenchyma, which is associated with SUDEP (14). Later we also described that repetitive convulsive stress acts as a general hypoxic condition that promotes P-gp overexpression associated with HIF-1α nuclear translocation in both the heart and brain parenchyma (12, 31). In the same way, in an independent experiment, we also showed that epileptic/hypoxic stress reduces the expression of the inward rectifier potassium channel 3.1 (kir3.1) (32), and we speculated that the inverse relationship between the expression of P-gp and Kir3.1 could be in part responsible for the depolarization of the cardiomyocyte membrane, and the aforementioned arrhythmia (13). Recent studies have shown that the mTOR pathway can also play an important role in SUDEP-associated cardiac disturbance in a HIF-1α-dependent manner (33).

In the present research, we show evidence that subconvulsive PTZ doses applied in a kindling model promoted cardiac hypoxic stress by increasing the HIF-1α stabilization in a similar way to that previously observed in SE (12). In addition, large hemorrhagic areas were observed. Some experimental and clinical studies have shown that intramyocardial hemorrhage often occurs in acute myocardial ischemia-reperfusion or infarction, resulting in iron accumulation and hemosiderin deposits that can be detected by magnetic resonance imaging with a long-lasting persistence (34–37). Likewise, we reported cardiomyocyte hypertrophy that could indicate overstimulation of the sympathetic system, as is also observed in chronic arterial hypertension, acute myocardial infarction, genetic cardiomyopathy, myocarditis, heart valve disease, thyroid disorders, obesity, diabetes, aging, among others (38). Interestingly, cardiomyocyte hypertrophy along with decreased gap junction and mitochondrial dysfunction was described as the most common cause of cardiac-related sudden death (39). Furthermore, the imbalance of iron metabolism and oxidative stress are also major factors for the evolution of cardiac hypertrophy, cardiac arrhythmia, and aging-associated pathological changes in the heart (40).

Additionally, convulsive/hypoxic stress will promote a tissue oxidative environment with ROS formation associated with free Fe^+2^, the Haber-Weiss and Fenton reactions, and iron overload as the main mechanism of cell death, called ferroptosis, that leads to tissue injury (24). This altered iron homeostasis allows uncontrolled entry and deposition of iron into different organs, including the heart, and leads to progressive damage with severe failure of affected organs (41, 42). Moreover, heme metabolism after tissue hemorrhage will produce hemosiderin accumulations for prolonged periods (43). Based on the different mechanisms described for cell death, ferroptosis is characterized by iron accumulation with deleterious effects on the functions of cells or organs.

**Figure 4:**
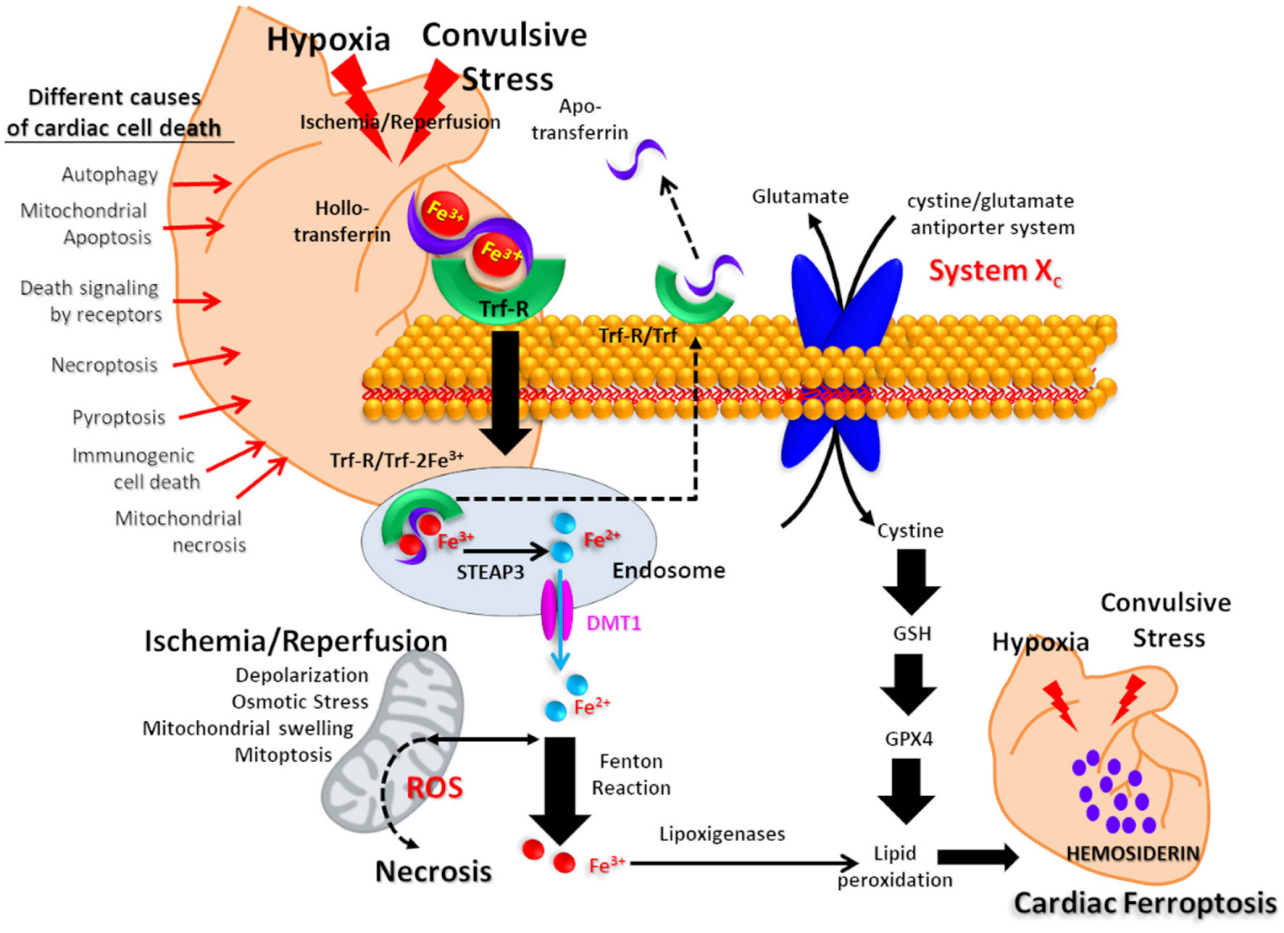
Proposed mechanism for cardiac iron overload related to postconvulsive hypoxia-ischemia. Iron transported by holotransferrin is recognized by the plasma membrane receptor (Trf-R). After endocytosis, Fe^3+^ is reduced to Fe^2+^ in endosomes by the metalloreductase six-transmembrane epithelial antigen of prostate 3 (Steap3) and released from endosomes by divalent metal transporter 1 (DMT1). After the Fenton reaction, iron promotes lipid peroxidation via the activation of lipoxygenases, resulting in hemosiderin accumulation. On the other hand, the axis composed of the cystine/glutamate antiporter system X_c_, Glutation (GSH) and Glutation peroxidase 4 (GPX4) is as a key regulator for tissue iron overload and ferroptosis. Other possible mechanisms of cardiomyocyte death are listed on the left side of the picture.

In this scenario, the hemosiderin deposits could be interpreted as an iron-scar indicating the site where a cell has died, after severe ischemia-reperfusion and oxidative stress processes. This complex mechanism could develop in the heart after repetitive convulsive/hypoxic stress. The finding of hemosiderin in the cardiomyocytes of our experimental SUDEP model suggests that ferroptosis has been present as a potential intrinsic mechanism that led to fatal cardiac arrhythmia (Figure 4) (44).

The accumulation of iron in the myocardium can result in a heart dysfunction known as “*Iron Overload Cardiomyopathy”* (IOC), which mainly described in certain inherited diseases related to alterations of iron/hemoglobin metabolism (hemochromatosis and thalassemia major, respectively), as well as secondary to a high rate of red blood cell transfusions (45). Furthermore, an adult patient with hereditary spherocytosis died after atrial tachycardia and cardiogenic shock, and the endomyocardial biopsy of the right ventricle revealed multiple myocardial hemosiderin deposits (46). Currently, IOC is assumed to be the most common cause of mortality in patients with secondary iron overload and is a major comorbidity in patients with genetic hemochromatosis (47, 48). IOC has been characterized by remodeling of the left ventricle (LV) with chamber dilation and reduced LV ejection fraction (LVEF) (49).

Recently, the concept of “Epileptic Heart” was defined as a heart and coronary vasculature damaged by chronic epilepsy as a result of repeated catecholamine surges and hypoxemia leading to electrical and mechanical dysfunction (50). In the “Epileptic Heart” syndrome several cardiological anomalies like ventricular hypertrophy, dilated cardiomyopathy, myocardial fibrosis, cardiomegaly, structural changes or dysfunction, higher levels of left ventricular stiffness, left ventricular filling pressures (by echocardiography), greater left atrial volume determined (by echocardiography), increased left ventricular filling pressure, end-systolic diameter, end-systolic volume, atrial fibrillation, and abnormal cardiac conduction have been reported. Interestingly, some of these same features were also described in the IOC.

Based on these data, we wonder if a potential “Epileptic Heart” might also exhibit iron overload in cardiomyocytes as described in the IOC syndromes. To our knowledge, this is the first experimental evidence showing increased iron overload in rat heart after induced SE, suggesting that repetitive severe seizures could lead to a fatal IOC-like syndrome.

## Potential Clinical Applications Related To This Research

In IOC, iron can be stored in myocytes in the form of ferritin, hemosiderin, and labile cell iron (free iron), the latter being the most active, but hemosiderin can be detected by imaging studies such as the cardiovascular magnetic resonance T2* (CMR-derived T2*). The increase in heart iron burden is inversely related to the relaxation time T2*. Thus, T2* values <20 ms, indicative of myocardial siderosis, have an inverse correlation with the LVEF, while T2* values <10 ms, indicative of severe iron overload, are associated with an increased annual risk of developing arrhythmias (51, 52). This non-invasive imaging method has revolutionized the clinical management of patients with hemoglobinopathies and other iron overload conditions.

CMR-derived T2* enables accurate diagnosis and quantification of myocardial and hepatic iron deposition, and hence the tailoring and monitoring of iron chelation therapy, thereby improving survival as in cases of thalassemia major (45, 53). Similarly, the topical application of the biological chelator lactoferrin to ulcerated legs of patients with ulcerative hemosiderinic dyschromia of chronic venous insufficiency, significantly reduced hemosiderin deposits, thus favoring the clearance of the iron deposition in the ulcerated legs as well as a complete ulcer scarring (54). Consistent with the possible use of CMR-derived T2* determining the iron heart, magnetic resonance imaging (MRI) with T1 mapping can distinguish fibrosis, edema, lipid accumulation, and iron and amyloid deposition (55). These changes included higher sympathetic drive, myocardial catecholaminergic toxicity, and cardiac fibrosis, and patients with refractory epilepsy may therefore have an arrhythmogenic phenotype. Cardiac imaging tools could help to identify cardiac injury as a biomarker of SUDEP risk.

## Data Availability Statement

The original contributions presented in the study are included in the article/supplementary material, further inquiries can be directed to the corresponding author/s.

## Ethics Statement

The animal study was reviewed and approved by CICUAL committee of the School of Medicine, University of Buenos Aires.

## Author Contributions

EA, ZD, EE, FM, AM, AL, and JA contributed to the experimental design and produce the results. EA, ZD, EE, FM, and JA analyzed the data. EA, AL, and JA wrote the text. All authors contributed to the article and approved the submitted version.

## Conflict of Interest

The authors declare that the research was conducted in the absence of any commercial or financial relationships that could be construed as a potential conflict of interest.

## References

[1] Verducci C Hussain F Donner E Moseley BD Buchhalter J Hesdorffer D. SUDEP in the North American SUDEP registry: the full spectrum of epilepsies. Neurology. (2019) 93:E227–36. 10.1212/WNL.0000000000007778 31217259 PMC6656646

[2] Langan Y Nashef L Sander JW Case-control study of SUDEP. Neurology. (2005) 64:1131–3. 10.1212/01.WNL.0000156352.61328.CB 15824334

[3] Shankar R Donner EJ McLean B Nashef L Tomson T. Sudden unexpected death in epilepsy (SUDEP): what every neurologist should know. Epileptic Disord. (2017) 19:1–9. 10.1684/epd.2017.0891 28218059

[4] Tomson T Nashef L Ryvlin P. Sudden unexpected death in epilepsy: current knowledge and future directions. Lancet Neurol. (2008) 7:1021–31. 10.1016/S1474-4422(08)70202-3 18805738

[5] Nashef L So EL Ryvlin P Tomson T. Unifying the definitions of sudden unexpected death in epilepsy. Epilepsia. (2012) 53:227–33. 10.1111/j.1528-1167.2011.03358.x 22191982

[6] Natelson BH Suarez RV Terrence CF Turizo R. Patients with epilepsy who die suddenly have cardiac disease. Arch Neurol. (1998) 55:857–60. 10.1001/archneur.55.6.857 9626779

[7] Devinsky O Kim A Friedman D Bedigian A Moffatt E Tseng ZH. Incidence of cardiac fibrosis in SUDEP and control cases. Neurology. (2018) 91:e55–61. 10.1212/WNL.0000000000005740 29858472 PMC6091880

[8] Lathers CM Schraeder PL. Autonomic dysfunction in epilepsy: characterization of autonomic cardiac neural discharge associated with pentylenetetrazol-induced epileptogenic activity. Epilepsia. (1982) 23:633–47. 10.1111/j.1528-1157.1982.tb05079.x 7173131

[9] Naggar I Lazar J Kamran H Orman R Stewart M. Relation of autonomic and cardiac abnormalities to ventricular fibrillation in a rat model of epilepsy. Epilepsy Res. (2014) 108:44–56. 10.1016/j.eplepsyres.2013.10.018 24286892

[10] Akyuz E Polat K Ates S Unalmis D Tokpinar A Yilmaz S. Investigating cardiac morphological alterations in a pentylenetetrazol-kindling model of epilepsy. Diagnostics. (2020) 10:388. 10.3390/diagnostics10060388 32526953 PMC7344915

[11] Auerbach DS Jones J Clawson BC Offord J Lenk GM Ogiwara I. Altered cardiac electrophysiology and sudep in a model of dravet syndrome. PLoS ONE. (2013) 8:e77843. 10.1371/journal.pone.0077843 24155976 PMC3796479

[12] Auzmendi J Buchholz B Salguero J Cañellas C Kelly J Men P. Pilocarpine-induced status epilepticus is associated with P-glycoprotein induction in cardiomyocytes, electrocardiographic changes, and sudden death. Pharmaceuticals. (2018) 11:21. 10.3390/ph11010021 29462915 PMC5874717

[13] Auzmendi J Akyüz E Lazarowski A. The role of P-glycoprotein (P-gp) and inwardly rectifying potassium (Kir) channels in sudden unexpected death in epilepsy (SUDEP). Epilepsy Behav. (2019). 10.1016/j.yebeh.2019.106590. [Epub ahead of print]. 31706919

[14] Auzmendi J Merelli A Girardi E Orozco-Suarez S Rocha L Lazarowski A Progressive heart P-glycoprotein (P-gp) overexpression after experimental repetitive seizures (ERS) associated with fatal status epilepticus (FSE). Is it related with SUDEP? Mol Cell Epilepsy. (2014) 1:43–51. 10.14800/mce.66

[15] Chen X Bao G Liu F. Inhibition of USP15 prevent glutamate-induced oxidative damage by activating Nrf2/HO-1 signaling pathway in HT22 cells. Cell Mol Neurobiol. (2020) 40:999–1010. 10.1007/s10571-020-00789-3 31933062 PMC11448803

[16] Kangralkar V Patil S Bandivadekar R Oxidative stress and diabetes: a review. Int J Pharm Appl. (2010) 1:38–45. 10.1176/pn.45.23.psychnews_45_23_048

[17] Oudit GY Sun H Trivieri MG Koch SE Dawood F Ackerley C. L-type Ca^2+^ channels provide a major pathway for iron entry into cardiomyocytes in iron-overload cardiomyopathy. Nat Med. (2003) 9:1187–94. 10.1038/nm920 12937413

[18] Tsushima RG Wickenden AD Bouchard RA Oudit GY Liu PP Backx PH. Modulation of iron uptake in heart by L-type Ca^2+^ channel modifiers: possible implications in iron overload. Circ Res. (1999) 84:1302–9. 10.1161/01.RES.84.11.1302 10364568

[19] Bodiga VL Thokala S Kovur SM Bodiga S. Zinc dyshomeostasis in cardiomyocytes after acute hypoxia/reoxygenation. Biol Trace Elem Res. (2017) 179:117–29. 10.1007/s12011-017-0957-7 28181174

[20] Knutson MD Non-transferrin-bound iron transporters. Free Radic Biol Med. (2019) 133:101–11. 10.1016/j.freeradbiomed.2018.10.413 30316781

[21] Das SK Wang W Zhabyeyev P Basu R Mclean B Fan D. Iron-overload injury and cardiomyopathy in acquired and genetic models is attenuated by resveratrol therapy. Sci Rep. (2015) 5:18132. 10.1038/srep18132 26638758 PMC4671148

[22] Pearson-Smith JN Patel M. Metabolic dysfunction and oxidative stress in epilepsy. Int J Mol Sci. (2017) 18:2365. 10.3390/ijms18112365 29117123 PMC5713334

[23] Lorigados L Morales LM Orozco-Suárez S Gallardo JM Díaz-Hung ML González ME Oxidative stress in pharmacoresistant epilepsy. (2016) 33:2101–7. Available online at: https://www.who.int/mediacen (accessed September 19, 2020).

[24] Yang WS Stockwell BR Ferroptosis: death by lipid peroxidation. Trends Cell Biol. (2016) 26:165–76. 10.1016/j.tcb.2015.10.014 26653790 PMC4764384

[25] Racine RJ. Modification of seizure activity by electrical stimulation II. Motor seizure. Electroencephalogr Clin Neurophysiol. (1972) 32:281–94. 10.1016/0013-4694(72)90177-0 4110397

[26] Whittaker P Hines FA Robl MG Dunkel VC Histopathological evaluation of liver, pancreas, spleen, and heart from iron-overloaded sprague-dawley rats*1,2. Toxicol Pathol. (1996) 24:558–63. 10.1177/019262339602400504 8923676

[27] Stewart M Kollmar R Nakase K Silverman J Sundaram K Orman R. Obstructive apnea due to laryngospasm links ictal to postictal events in SUDEP cases and offers practical biomarkers for review of past cases and prevention of new ones. Epilepsia. (2017) 58:e87–90. 10.1111/epi.13765 28464295

[28] Nakase K Kollmar R Lazar J Arjomandi H Sundaram K Silverman J. Laryngospasm, central and obstructive apnea during seizures: defining pathophysiology for sudden death in a rat model. Epilepsy Res. (2016) 128:126–39. 10.1016/j.eplepsyres.2016.08.004 27835782

[29] Mooney S Kollmar R Gurevich R Tromblee J Banerjee A Sundaram K. An oxygen-rich atmosphere or systemic fluoxetine extend the time to respiratory arrest in a rat model of obstructive apnea. Neurobiol Dis. (2020) 134:104682. 10.1016/j.nbd.2019.104682 31759134

[30] Ryvlin P Nashef L Lhatoo SD Bateman LM Bird J Bleasel A. Incidence and mechanisms of cardiorespiratory arrests in epilepsy monitoring units (MORTEMUS): a retrospective study. Lancet Neurol. (2013) 12:966–77. 10.1016/S1474-4422(13)70214-X 24012372

[31] Merelli A Czornyj L Lazarowski A. Erythropoietin as a new therapeutic opportunity in brain inflammation and neurodegenerative diseases. Int J Neurosci. (2015) 125:793–7. 10.3109/00207454.2014.989321 25405533

[32] Akyüz E Mega Tiber P Beker M Akbaş F. Expression of cardiac inwardly rectifying potassium channels in pentylenetetrazole kindling model of epilepsy in rats. Cell Mol Biol. (2018) 64:47–54. 10.14715/cmb/2017.64.15.8 30672436

[33] Sharma S Mazumder AG Rana AK Patial V Singh D. Spontaneous recurrent seizures mediated cardiac dysfunction *via* mTOR pathway upregulation: a putative target for SUDEP management. CNS Neurol Disord - Drug Targets. (2019) 18:555–65. 10.2174/1871527318666190801112027 31368880

[34] Cokic I Wawrowsky K Kali A Yang H-J Tang R Francis J Acute and stable ischemic heart disease myocardial hemorrhage after coronary ischemia-reperfusion leads to an iron-mediated, self-perpetuating loop of foam cell and ceroid accumulation. J Am Coll Cardiol. (2017) 69(Suppl. 11):197 10.1016/S0735-1097(17)33586-6

[35] Kumar A Green JD Sykes JM Ephrat P Carson JJL Mitchell AJ. Detection and quantification of myocardial reperfusion hemorrhage using T2 (*)-weighted CMR. JACC Cardiovasc Imaging. (2011) 4:1274–83. 10.1016/j.jcmg.2011.08.016 22172784

[36] Van Den Bos EJ Baks T Moelker AD Kerver W Van Geuns RJ Van Der Giessen WJ. Magnetic resonance imaging of haemorrhage within reperfused myocardial infarcts: possible interference with iron oxide-labelled cell tracking? Eur Heart J. (2006) 27:1620–6. 10.1093/eurheartj/ehl059 16751204

[37] Anderson LJ Westwood MA Holden S Davis B Prescott E Wonke B. Myocardial iron clearance during reversal of siderotic cardiomyopathy with intravenous desferrioxamine: a prospective study using T2* cardiovascular magnetic resonance. Br J Haematol. (2004) 127:348–55. 10.1111/j.1365-2141.2004.05202.x 15491298

[38] Maillet M Van Berlo JH Molkentin JD. Molecular basis of physiological heart growth: fundamental concepts and new players. Nat Rev Mol Cell Biol. (2013) 14:38–48. 10.1038/nrm3495 23258295 PMC4416212

[39] Mansueto G Benincasa G Capasso E Graziano V Russo M Niola M. Autoptic findings of sudden cardiac death (SCD) in patients with arrhythmogenic ventricular cardiomiopathy (AVC) from left ventricle and biventricular involvement. Pathol-Res Pract. (2020) 216:153269. 10.1016/j.prp.2020.153269 33176260

[40] Kumar V Santhosh Kumar TR Kartha CC. Mitochondrial membrane transporters and metabolic switch in heart failure. Heart Fail Rev. (2019) 24:255–67. 10.1007/s10741-018-9756-2 30535838

[41] Buja LM Roberts WC. Iron in the heart. Etiology and clinical significance. Am J Med. (1971) 51:209–21. 10.1016/0002-9343(71)90240-3 5095527

[42] Hentze MW Muckenthaler MU Andrews NC. Balancing acts: molecular control of mammalian iron metabolism. Cell. (2004) 117:285–97. 10.1016/S0092-8674(04)00343-5 15109490

[43] Felberg RA Grotta JC Shirzadi AL Strong R Narayana P Hill-Felberg SJ. Cell death in experimental intracerebral hemorrhage: the “black hole” model of hemorrhagic damage. Ann Neurol. (2002) 51:517–24. 10.1002/ana.10160 11921058

[44] Del Re DP Amgalan D Linkermann A Liu Q Kitsis RN. Fundamental mechanisms of regulated cell death and implications for heart disease. Physiol Rev. (2019) 99:1765–817. 10.1152/physrev.00022.2018 31364924 PMC6890986

[45] Kremastinos DT Farmakis D. Iron overload cardiomyopathy in clinical practice. Circulation. (2011) 124:2253–63. 10.1161/CIRCULATIONAHA.111.050773 22083147

[46] Fujino T Inoue S Katsuki S Higo T Ide T Oda Y. Fatal cardiac hemochromatosis in a patient with hereditary spherocytosis. Int Heart J. (2018) 59:427–30. 10.1536/ihj.17-160 29563373

[47] Murphy CJ Oudit GY. Iron-overload cardiomyopathy: pathophysiology, diagnosis, and treatment. J Card Fail. (2010) 16:888–900. 10.1016/j.cardfail.2010.05.009 21055653

[48] Pennell DJ Udelson JE Arai AE Bozkurt B Cohen AR Galanello R. Cardiovascular function and treatment in β-thalassemia major: a consensus statement from the american heart association. Circulation. (2013) 128:281–308. 10.1161/CIR.0b013e31829b2be6 23775258

[49] Albakri A Iron overload cardiomyopathy: a review of literature on clinical status and meta-analysis of diagnostic and clinical management using iron chelators. Intern Med Care. (2018) 2 10.15761/IMC.1000117

[50] Verrier RL Pang TD Nearing BD Schachter SC. The epileptic heart: concept and clinical evidence. Epilepsy Behav. (2020) 105:106946. 10.1016/j.yebeh.2020.106946 32109857

[51] Kirk P Roughton M Porter JB Walker JM Tanner MA Patel J. Cardiac T2* magnetic resonance for prediction of cardiac complications in thalassemia major. Circulation. (2009) 120:1961–8. 10.1161/CIRCULATIONAHA.109.874487 19801505 PMC2784198

[52] He T. Cardiovascular magnetic resonance T2* for tissue iron assessment in the heart. Quant Imaging Med Surg. (2014) 4:407–12. 10.3978/j.issn.2223-4292.2014.10.05 25392825 PMC4213428

[53] Modell B Khan M Darlison M Westwood MA Ingram D Pennell DJ Improved survival of thalassaemia major in the UK and relation to T2* cardiovascular magnetic resonance. J Cardiovasc Magn Reson. (2008) 10:42 10.1186/1532-429X-10-42 18817553 PMC2563008

[54] Brizzio E Castro M Narbaitz M Borda N Carbia C Correa L Ulcerated hemosiderinic dyschromia and iron deposits within lower limbs treated with a topical application of biological chelator. Veins Lymphat. (2012) 1:18–26. 10.4081/vl.2012.e6

[55] Fialho GL Wolf P Walz R Lin K. Epilepsy and ultra-structural heart changes: the role of catecholaminergic toxicity and myocardial fibrosis. What can we learn from cardiology? Seizure. (2019) 71:105–9. 10.1016/j.seizure.2019.07.002 31306872

